# General practitioners’ views towards management of common mental health disorders: Τhe critical role of continuing medical education

**DOI:** 10.1186/s12875-023-02017-5

**Published:** 2023-03-04

**Authors:** Ilias Papachristopoulos, Eleni Sazakli, Michalis Leotsinidis

**Affiliations:** 1 Lab of Public Health, Medical School, University of Patras, GR-26504 Patras, Greece; 2 Primary Health Care Centre of Kleitoria, Achaia, Greece

**Keywords:** Common mental disorders, General practitioners, Mental health education, Primary care, Depression, Anxiety

## Abstract

**Background:**

The disability burden of common mental health disorders is enormous and should be faced at the first point of contact in the healthcare system. General Practitioners (GPs) are called to recognize, diagnose, and manage patients with mental health disorders, a task which is not always addressed successfully. The study aims at examining the relationship between mental health education of GPs and self-reported opinions on the care they provide to patients with mental disorders in Greece.

**Methods:**

A questionnaire investigating GPs' viewpoints regarding diagnostic methods, referral rates and overall management of patients with mental disorders, and how these are impacted by their education on mental health, was employed, in a randomly selected sample of 353 GPs in Greece. Suggestions and proposals about improvement of ongoing mental health training, along with organizational reforming were also recorded.

**Results:**

Received Continuing Medical Education (CME) is characterized as insufficient by 56.1% of the GPs. More than half of the GPs participate in clinical tutorials and mental health conferences once per three years or less. The level of educational score on mental health is associated positively with decisiveness on management of patients and increases self-confidence. A percentage of 77.6% states knowledge of the appropriate treatment and 56.1% agree to initiate treatment without referring to a specialist. However, low to moderate self-confidence about diagnosis and treatment is stated by 47.5%. According to GPs, critical points for improving mental health primary care are the liaison psychiatry and high degree of CME.

**Conclusion:**

Greek GPs are calling for focused and continuing medical education, in the field of psychiatry, along with essential structural and organizational reforming of the healthcare system, including an efficient liaison psychiatry.

**Supplementary Information:**

The online version contains supplementary material available at 10.1186/s12875-023-02017-5.

## Background

Common mental disorders (CMDs), also referred as neurotic disorders, include different types of depression and anxiety disorders and can cause intense emotional distress, usually without affecting the cognitive function of individuals [1]. The global estimate of CMDs prevalence was 970,812,400 for 2017 (95% uncertainty interval 923,455,400 to 1,020,930,600) [2]. The overwhelming part of mental disorders burden (92.6%) is non-fatal, so disability indicators reflect better the true impact of these disorders [3]. For more than 30 years, mental disorders account for more than 14% of age-standardized years lived with disability (YLDs) [2]. Additionally, the barrier of stigma and avoidance of seeking treatment may contribute to significant under-reporting, especially in low- and lower-middle-income countries [3]. Therefore, the need for correct diagnosis and treatment of patients with mental disorders is of crucial importance.

As primary care is the first point of contact in the healthcare system, most patients, in order to settle the mental disorder they suffer from, are more likely to address a Primary Health Care (PHC) structure and a General Practitioner (GP), rather than a psychiatrist or a specialized mental health center [4, 5]. This is due to various reasons, including the greater intimacy of the patient with a GP, patient's fear of being stigmatized as mentally ill and the long waiting times required to be examined by a mental health professional [4, 5]. Moreover, the GP is often able to develop a long-term, curative and trusting relationship with patients and be aware of their family background and psychosocial status, a relationship that may be quite onerous to be established with a doctor of another specialization [6].

Although their role is very important in the outcome of a patient's disease, it has been proved that primary care physicians often fail to recognize, diagnose and treat properly mental diseases [7–9]. It should be noted that misdiagnosis refers not only to under-diagnosis and under-treatment of CMDs, but the opposite as well, i.e. cases of patients being characterized as mentally ill without meeting the appropriate criteria, thus receiving pointless medical treatments [10]. Although in most cases GPs can exclude depression in people who do not suffer from it (i.e. good specificity), they can only recognize about half of those suffering from depression (i.e. low sensitivity) [8, 11]. The untreated cases include not only mild and often self-limiting mental disorders, but also severe cases, that are in urgent need of receiving treatment [12]. The ability to discern severity of mental disorders and adapt therapeutic scheme accordingly, is another skill that GPs should develop [13].

Causes of under-recognition and, consequently under-treatment, of CMDs in the community are multi-factorial and include barriers associated with the healthcare system and issues related to both patients and physicians [7, 14]. System barriers involve limited in-service training in mental health care, inefficient performance of liaison psychiatry, lack of standardized methods for detecting, treating and continuing care for mentally ill and inadequate incentives to GPs for the treatment of patients with mental disorders. From the patient's point of view, the main barriers reported are resistance to accept diagnosis of a mental health problem, especially when somatic symptoms coexist, access constraints, fear of stigmatization, beliefs that a GP is not the right person to talk to, and lack of health insurance. Physicians' factors include poor education and inadequate training, wrong beliefs about mental illness, discomfort in attending such patients, limited time and not regarding mental illnesses management as their primary role [14, 15].

In Greece, in order to specialize in General Practice, a medical physician should attend a 5-year residency program that includes continuing and structured rotation through hospital and ambulatory care settings and education seminars. Training in psychiatric clinics is mandatory for GPs and lasts three months. In most of the other European countries, there is about three (e.g. UK, France, Italy) to five years (e.g. Germany, Denmark, Sweden) of post-graduate training to specialize in family medicine, while training in psychiatry is in some countries mandatory (e.g. Sweden) and in others optional (e.g. UK, Italy) and lasts for 3–6 months [16]. Beyond good education and adequate training, the issue of the medical density of GPs has a great influence on the provided health care and the patient's level of satisfaction, as it affects the available time of the GP to spend with patients. While Greece had the highest per capita rate of licensed physicians among EU Member States (6.1 per 1000 inhabitants in 2018), it reported the lowest share of general practitioners, i.e. one in 20 physicians versus one in five on average across EU countries [17]. While a single number cannot reflect the ideal proportion of GPs for a population to be adequately served, it has been reported that one practitioner per 1,800 inhabitants is considered sufficient [18]. The GPs shortage in Greece (one GP per 3570 inhabitants) entails heavy workload for Greek GPs, a fact that may further add to ineffective treatment and high referral rates of patients with common mental disorders.

The aim of the present study was to investigate the perceptions, attitudes and opinions of GPs on the care they provide to patients with CMDs in Greece, and identify how these are associated with mental health education. In addition, GPs' suggestions regarding improvement of management of mental health disorders into the primary care were recorded.

## Methods

### Study design

A cross-sectional survey was undertaken within primary healthcare settings in Greece.

### Recruitment of study subjects

The total number of GPs in Greece in 2017 was 3,054 [19]. The ideal sample size, allowing a margin of error ± 5% with 95% confidence level, was calculated at 342 participants. In order to accomplish that and considering previous reported response rates of 54—60% in similar studies [5, 20, 21], the 20% of the total Greek GPs was set as a target sample size. An announcement of the survey briefly describing the study, its objectives and procedures, and the link of the electronic platform with the questionnaire was mailed out to the 60 prefectural medical associations in Greece, with the request to be notified to the 20% of their registered GPs, by random selection. GPs were assured that participation was voluntary and the confidentiality and anonymity would be retained. Moreover, personal contact data of the first author was given, in case that clarifications were needed. Three electronic reminders were re-sent to the medical associations, on a monthly basis. The recruitment period lasted four months. Registration of the results was recorded in an electronic database.

### Data collection

Data were collected using a self-administered anonymous electronic questionnaire that was designed for GPs. The questionnaire consisted of 32 questions, divided into four sets of questions. The first set concerned demographic data, occupational status, and years of experience. The second set contained 15 Likert-scale questions inquiring the degree of agreement in statements concerning physicians' education on mental health, adequacy of Continuing Medical Education (CME), diagnostic methods for mental disorders, knowledge of proper treatment, decisions on following or referring a patient, collaboration with psychiatrists and mental health centers, observation of a patient after referral and adequacy of healthcare system provisions. Two scaling questions, from zero to ten, concerning self-confidence in diagnosis and treatment, and rates of referrals, and one closed question about actions in a case of no response to treatment, were also included in this set. In the third set of questions, GPs were asked to fill in three open-ended questions about frequency of occurrence of most CMDs and decisions on which mental disorders they treat and which they refer to a specialist. Finally, the fourth set comprised of three open-ended questions about GPs’ expectations on the education offered by public institutions, suggestions on improving healthcare system provisions and opinions on the proper integration of mental health in the PHC. The full-questionnaire and details about the pilot survey are presented in the Supplement.

### Data analysis

Descriptive statistics were computed to summarize demographic data of GPs. Group proportions were calculated for categorical variables. Differences in proportions were tested using the x^2^ test or the Fisher's exact test. Pearson r correlation coefficient was calculated to assess bivariate associations. Differences in means between two or more groups, after testing for normality, were examined with the t-test or the one-way ANOVA test, respectively. Conventional factor analysis using Varimax rotation was performed on the correlation matrix of the questions, to identify observable variables that were inter-correlated. Following factor analysis, four ordinal questions related to GPs' education on mental health were summed up and converted to a continuous numerical variable. Each response in the Likert scale took a numerical value from 1 to 5, and the sum of all four responses was calculated for each participant. In that way, a new scale variable named "Educational Score" was created.

General linear models were applied to assess the effect of the "educational score" of GPs on the self-reported management of patients, as reflected in their answers. Univariate logistic regression models were employed to estimate the relationship between GPs' level of experience and decisions regarding following/referral of mental disorders. Statistical processing was performed with IBM SPSS v.24 (IBM Corp., Armonk, NY, USA).

## Results

### Study participants

In total, 353 GPs participated in the study (response rate: 57.8%). The demographic characteristics of the participants are presented in the Supplement (Table S1).

### Continuing medical education (CME) of GPs on mental disorders

The self-reported CME of GPs in the field of mental health is presented in Table 1.

**Table 1:** Self-reported Continuing Medical Education (CME) of GPs on mental disorders

	*N* = *353*	At least twice per year	Once per year	Every two years	Every three years	Less than once in three years	Never	No response
Q_1_	%	8.8	27.2	16.7	10.2	31.2	5.7	0.3
Q_2_		5.9	19.5	12.7	12.7	34.3	14.7	0.0
		Once a week	Once a month	2–3 times per year	Once per year	Rarely	Never	No response
Q_3_	%	11.3	25.5	38.2	8.2	15.0	1.7	0.0
Q_4_		13.6	33.7	31.4	11.3	7.9	1.4	0.6

Q_1_: I participate in psychiatric seminars/conferences for my continuous education on mental disorders

Q_2_: I participate in clinical training psychiatric courses so as to practice my skills on mental disorders' management

Q_3_: I study scientific articles, contemporary research, and psychiatric textbooks to keep pace with most common mental disorders I may need to manage

Q_4_: I get informed on the treatment of most common mental disorders as far as therapeutic effects, pharmacokinetics and drugs' side effects are concerned

It is observed that 47.1% of the responders declare that they participate in psychiatric conferences (Question 1, Q_1_) and 61.7% in clinical tutorials (Q_2_) once every three years or less often. On the other hand, on a monthly basis or more often, 36.8% of them affirm the update of their knowledge on psychiatry through scientific articles and textbooks (Q_3_), and 47.3% are keeping informed about therapeutic strategies, pharmacokinetics and reported adverse effects of administered drugs (Q_4_).

### GP’s self-assessment of CMDs management

Table 2 presents the GPs’ self-assessment on the adequacy of CME on mental health, the diagnostic methods they use to detect a mental disorder, and the patients' management they follow.

**Table 2:** Methods of diagnosis and management of a patient with mental disorder

Question	Description	Completely disagree	Disagree	Rather disagree	Rather agree	Agree	Completely agree	No response
		% (*N* = 353)						
Q_5_	My CME in mental health is adequate	4.0	18.1	34.0	25.2	13.3	5.4	0.0
Q_6_	Diagnosis is based on psychometric techniques	2.0	10.8	18.4	32.3	28.9	7.6	0.0
Q_7_	Diagnosis is based on psychiatric interview and on DSM-V	1.7	7.4	9.1	31.7	38.2	11.9	0.0
Q_9_	I am aware of the proper treatment	0.3	7.4	14.4	43.9	30.3	3.4	0.3
Q_10_	After diagnosis, I refer directly to the psychiatrist	9.9	27.5	30.6	17.6	8.8	5.7	0.0
Q_11_	After diagnosis, I recommend treatment initiation without referral to a specialist	5.4	15.0	23.5	30.9	19.8	5.4	0.0
Q_12_	I follow up only if the patient has received treatment and precise instructions by a psychiatrist	6.5	22.9	30.6	18.7	17.6	3.7	0.0
Q_13_	I am aware of the mental health centres and services, so as to be able to refer	0.8	1.7	4.5	14.2	50.7	27.8	0.3
Q_15_	I co-operate with psychiatrists and mental health centres, to decide on patient management	2.8	9.3	12.2	25.8	37.4	12.5	0.0
Q_16_	I am provided with all appropriate conditions to treat a patient with a psychiatric disorder, from diagnosis to recovery	11.6	33.1	30.0	14.7	8.5	2.0	0.0
		Always	Very often	Often	Some- times	Rarely	Never	
Q_14_	After referral to a specialist, I contact and get informed about diagnosis and possibly joint follow-up of the patient	21.5	23.5	22.9	22.4	7.1	2.5	0.0

It is deduced that 56.1% do not consider their CME to be adequate to treat a patient with mental disorder (Q_5_). Diagnosis is based on psychiatric interview and DSM-V criteria by 81.8% of the responders (Q_7_), while psychometric tools (tests and questionnaires) are preferred by the 68.8% (Q_6_). A percentage of 77.6% states that they are aware of the proper treatment (Q_9_) and 56.1% agree to initiate treatment without referring to a specialist (Q_11_). On the other hand, 32.1% will refer directly to a psychiatrist, without any intervention of their own (Q_10_). Among all, 40.0% state to follow-up, only if the patient has received treatment and accurate instructions by a psychiatrist (Q_12_).

In case a patient needs referral, the majority (92.6%) declare awareness of the appropriate mental health centers (Q_13_) and 75.6% claim to collaborate with these centers for the joint treatment of the patient, if required (Q_15_). Most of them (67.9%) state they keep contact and get informed about the progress of their patient, after referral to a psychiatrist (Q_14_). In case of an ineffective treatment, 68.5% would refer to a specialist and only 30.3% would modify their initial therapeutic scheme and wait for the outcome (Q_17_). The majority of GPs (74.7%) consider they are provided with inadequate conditions for appropriately diagnosing, treating and following a psychiatric patient (Q_16_).

Regarding self-confidence of GPs about diagnosis and treatment of mental disorders, it was assessed by a question (Q_8_), in which respondents ranked their answers on a numerical scale from zero to ten, where zero indicates "not at all" confidence and ten "absolute" confidence. Self-confidence level was higher in men (mean 6.44, s.d. 1.86) than in women (mean 5.98, s.d. 2.09), (t-test, *p* = 0.029). It also correlated with years of experience (Pearson r = 0.135, *p* = 0.011). Further categorization revealed that a percentage of 52.5% was "quite to very" confident about diagnosis and treatment of mental disorders (rank 7 to 10), another 30.5% declared "moderate" confidence (rank 5 to 6), while 17% felt "less or not at all" confident (rank 0 to 4).

Factor analysis of the Likert-scale questions extracted four factors, that explained 58% of the total variance. The first factor was mainly related with opinions about diagnostic methods (Q_6_, Q_7_), knowledge of treatment (Q_9_), healthcare system provisions (Q_16_), self-confidence level (Q_8_), and self-assessed adequacy of CME (Q_5_), with the latter presenting loading on the second factor as well. The four questions regarding training on mental health (Q_1_-Q_4_) were associated almost exclusively with the second factor, reflecting the educational status of GPs on mental health. Questions about the attitudes of GPs towards referrals to specialists after diagnosis (Q_10_, Q_11_, Q_12_, Q_17_) were loaded on the third factor. Finally the fourth factor was related with questions regarding the cooperation and consultation of GPs with psychiatrists (Q_13_, Q_14_, Q_15_).

"Educational score" that was created from the four training questions (Q_1_-Q_4_), which were loaded on one factor, was used to assess whether mental health education is associated with GPs' self-confidence and attitudes towards management of mentally ill patients. The score did not differ between men (mean 11.17, s.d. 4.43) and women (mean 10.62, s.d. 4.23) (t-test, *p* = 0.238), between experienced (years of employment > 5, mean 11.22, s.d. 4.45) and novice GPs (years of employment < 5, mean 10.47, s.d. 4.15) (t-test, *p* = 0.113) and among setting of employment (private practice, health centre, regional medical centre and hospital), (one-way ANOVA, *p* = 0.117). The "educational score" correlated with the self-confidence level of the GPs (Pearson r = 0.408, *p* < 0.001).

**Fig. 1:**
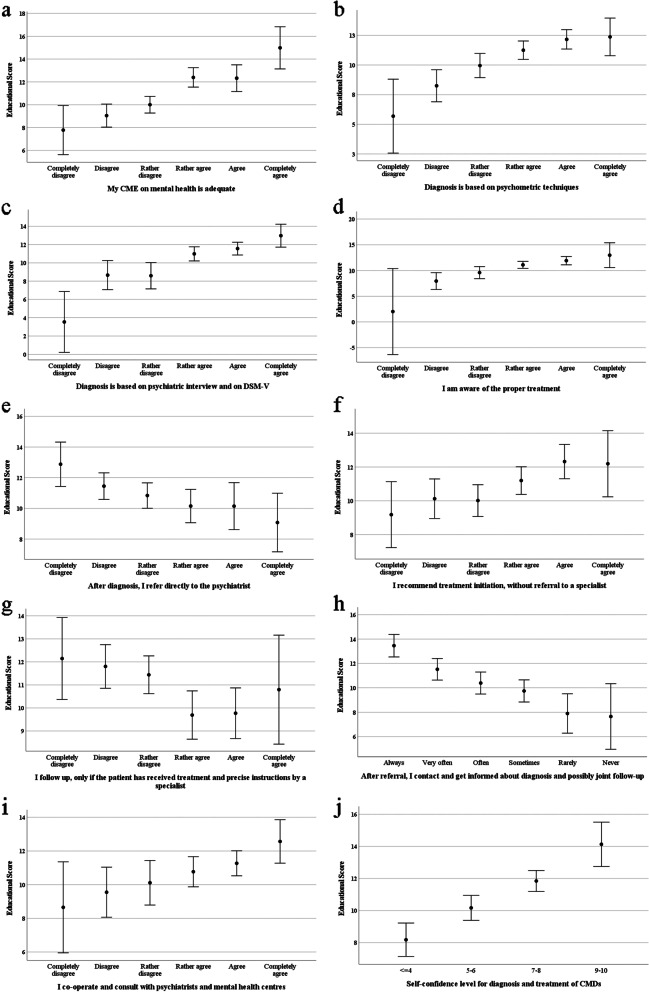
Associations of educational score with levels of factors concerning patients' management: **a**) adequacy of CME in mental health, **b**) diagnosis based on psychometric tests, **c**) diagnosis based on psychiatric interview and DSM-V criteria, **d**), awareness of the proper treatment, **e**) direct referral to a specialist, **f**) treatment initiation without referral to a specialist, **g**) following patients, only if treatment and instructions are given by a specialist, **h**) observing patient progress, **i**) cooperation and consultation with specialists, and **j**) self-confidence level for diagnosis and treatment. Error bars present mean values ± 2 standard errors

Univariate analysis of variance was conducted to examine whether the "educational score" of the GPs is associated with attitudes towards diagnosis, treatment, and referrals of patients. All models were controlled for years of experience. In general, the higher the educational score of GPs is, the more involved they become in diagnosis, follow-up and treatment of patients with mental disorders. The results of the General Linear models are presented in detail in Fig. 1. Covariate (years of experience) is evaluated at the value of 8.4.

### Current management of mental disorders as expressed by the GPs

Frequency of occurrence of the most CMDs, according to the GPs' statements (Q_18_), is presented in Table 3, in descending order.

**Table 3:** Rank-frequency distribution of Mental Disorders

	Order of frequency						
	1st	2nd	3rd	4th	5th	6th	Total
	% (*N* = 353)						
Depression	49.0	36.8	9.6	1.1	0.3	0.0	***96.9***
Anxiety Disorders	42.5	30.3	6.5	1.4	0.0	0.0	***80.7***
Psychotic Disorders	1.4	2.8	16.7	14.2	18.1	9.3	***62.6***
Bipolar Disorders	0.6	3.7	9.3	11.9	11.0	4.5	***41.1***
Panic Disorders	2.5	8.8	14.7	5.4	1.1	0.0	***32.6***
Organic Mental Disorders	0.3	0.6	5.9	5.7	3.1	1.7	***17.3***
Dementia	1.1	2.5	4.0	3.1	2.3	0.3	***13.3***
Obsessive–Compulsive Syndrome	0.0	1.1	1.7	3.1	3.1	1.7	***10.8***
Sleep Disorders	1.1	1.4	3.4	1.4	0.8	0.0	***8.2***
Alcoholism-Dependence Syndrome	0.0	0.3	2.0	5.9	2.8	1.1	***12.2***
Somatoform Disorders	0.3	1.7	1.7	2.3	0.3	0.6	***6.8***
Phobic Anxiety Disorders	0.0	0.6	2.3	3.1	1.4	0.8	***8.2***
Other	1.4	4.0	9.6	7.6	5.9	4.2	***32.9***

Depression and anxiety disorders were the most frequent, being ranked at the first and second place, respectively. Moreover 62.6% of GPs stated visits by patients with psychotic disorders. No difference in the ranking of mental disorders was observed between novice (years of experience 0–5, *N* = 139, 39.4%) and experienced GPs (years of experience > 5, *N* = 214, 60.6%) (Fisher exact tests, *p* > 0.050).

In the question about referral rate of patients with mental disorders to a specialist (Q_21_), the average rate was 41.0%. Figure S1 depicts the distribution of referrals among GPs, on a percentage basis. It is observed that 39% of GPs stated they refer at least half of the cases they meet. According to GPs statements, 85.0% are treating depression and 72.2% anxiety. Only 3.4% of depressive patients are referred, while anxiety disorder is not reported by any GP as a disease being referred to a psychiatrist. Experienced GPs (> 5 years of employment) had 2.09 higher odds (95% C.I.: 1.16—3.76) to follow depression, 2.27 higher odds (95% C.I.: 1.08—4.78) to follow organic mental disorders, and 1.58 higher odds to refer psychotic disorders (95% C.I.: 1.03—2.43) than their inexperienced colleagues, while novice GPs (< 5 years experience) had 2.10 higher odds (95% C.I.: 1.03—4.29) to refer a patient in case of doubtful diagnosis.

Table S2 presents the proportion of mental disorders that GPs stated to refer to a specialist (Q_20_) and those that GPs stated to treat (Q_19_), in a descending order.

### Educational score and self-confidence profiles of GPs related to following or referring distinct CMDs

The "educational score" was compared between participants who declared to undertake care of or refer distinct mental disorders. The mean "educational score" was higher in GPs stating they follow depression (t-test, 11.14 vs 9.74, *p* = 0.030) and dementia (12.79 vs 10.73, *p* = 0.008) and they refer psychotic disorders (11.39 vs 10.41, *p* = 0.036), and lower in GPs reporting they refer all mental disorders (9.20 vs 11.06, *p* = 0.039). Similar comparisons regarding the self-confidence level, showed that it was higher in GPs stating they follow depression (6.35 vs 5.60, *p* = 0.011), anxiety disorders (6.36 vs 5.91, *p* = 0.052), organic mental disorders (6.86 vs 6.15, *p* = 0.030), dementia (6.91 vs 6.17, *p* = 0.027) and lower in those reporting they refer depressive patients (5.00 vs 6.28, *p* = 0.027), or all mental disorders (5.00 vs 6.33, *p* = 0.014).

### GPs’ suggestions for improving CMD management

In the last three open-ended questions, GPs expressed their proposals about ways of improving their education on mental health problems, of providing facilitators by the healthcare system and of integrating mental health into primary care. Regarding education, the majority of GPs (75.9%) advocated various methods of continuing specialized learning, with the top two preferences being mental health seminars (45.9%), and specialized schools (24.5%) (Figure S2). Practical training delivered by specialized physicians or mental health centers, such as clinical tutorials, workgroups and case reports, were preferred by 41.8%. A percentage of 20.4% advocated both theoretical learning and practical training.

As far as the healthcare system is concerned, they would like a more effective liaison psychiatry (59.1%) and better organization (34.7%) (Figure S3). In accordance, GPs consider education (47.0%) and liaison psychiatry (37.9%) as the preferred ways to integrate mental health into primary care successfully (Figure S4).

## Discussion

This study demonstrates that more than half (56.1%) of the GPs in our sample consider their CME inadequate to treat mental health disorders, while 17% declare "little or not at all" confidence in diagnosis and treatment of mental problems. On the other hand, the more educated on mental health they are, the higher their involvement in attendance and proper management of mentally ill patients, a finding that reflects the importance of focused CME on mental health.

We found that 82% of the GPs declare they are aware of and use diagnostic criteria for the detection of CMDs, but only 58.2% of them feel "quite to very" confident about their diagnosis. In another study with a similar percentage (84%) of criteria knowledge, only 41% were actually based on them to make a diagnosis. According to these authors, individual skills of the physician, characteristics of the patient and effective counseling, seem to play a major role in diagnosis [20].

Regarding therapeutic approach, the percentage of physicians that prescribe medication (56.1%) is significantly lower than that reporting awareness (77.8%) of the appropriate medication for the disease they have diagnosed. In a cross-sectional study from five southeastern European countries, even though 40.9% of GPs state that can treat depression with medication, 79.4% believe patients in need of antidepressants should do better if referred to a psychiatrist [22]. A questionable therapeutic approach of GPs is highlighted in an Italian study, as 35% from those who initiated treatment, were in fact false-positive cases of depression [10]. The above mentioned results indicate a mixed literature on the comfort and accuracy of GPs in practicing mental healthcare, that are variable by country, level of education and structure of the healthcare system.

The referral rate observed in our study (41.0%) is comparable with two USA studies reporting that GPs refer 36–37% of mentally ill patients [20, 23]. In Germany, where only 7.5% stated they prefer to refer patients to a specialist, the actual percentage of referrals reached the 38.6% of depressive patients, of which 21.9% were truly positive and another 16.7% were falsely diagnosed with other psychiatric disorders [8]. In this study, specific obstacles faced by primary care doctors were identified, including time limitation and high frequency visits, which may be overcome by structural changes in the healthcare system and reimbursement schemes [8]. In France, where two cross-sectional surveys with the same methodology were carried out 10 years apart, GPs' referral rates to a specialist increased from 9.7% in 2003 to 14.7% in 2013 [24]. These fluctuations in referral rates may be the result of structural and organizational differences in healthcare systems of the countries, as well as differences in the education and training of GPs on mental health, even in countries with advanced healthcare systems.

Another issue of extreme importance is the appropriate identification of mental disorders for referral to the specialist. Previous studies have clarified that a GP should at least be able to follow common mental disorders, i.e. depression and anxiety, and recognize serious mental disorders, i.e. schizophrenia, bipolar disorder, in order to refer them [25, 26], or should refer patients when the severity of the disease requires treatment by a specialist. In our study, GPs stated that psychosis, bipolar affective disorder, severe depressive episode, comorbid mental illness, and no response to treatment are the main causes of referrals. On the other hand CMDs, such as neurotic, mood and anxiety disorders, are usually treated by GPs in their office, as expected by their specialty. Even though GPs stated they comply with the guidelines about referrals, their self-confidence does not reflect certainty on their practice. All these obstacles identified in our study, i.e. accuracy in diagnosis, treatment and referrals of mental disorders in the primary healthcare can be counteracted by reinforced and focused mental health education [4], supported by primary care liaison which enables knowledge transfer and shared care by GPs and mental health specialists [25].

The crucial role and necessity of CME of GPs in the field of psychiatry, has been highlighted in other studies [4, 8, 27] and in core competencies described for general practitioners [28]. We found that the 'Educational Score' greatly influences GPs' responses concerning diagnosis and treatment of CMDs, in consistency with a European study reporting that GPs feel more confident after participating in a training program compared to the baseline [29]. Similarly, in two Australian studies, GPs who had received mental health training were at higher odds of being more confident in diagnosis and treatment of mental disorders [21, 30]. However, training courses not only increase self-confidence, but also improve actual capabilities, given that in Germany, training courses resulted in two times higher probability of correctly recognising a patient [8].

Worries and desire of GPs for efficient education on mental health are complemented with their suggestions about structural and organizational reforms of the National Health Care System. Close collaboration with mental health specialists, in both training and practice, is of eminent importance. In order to guide an efficient support of mental disorders at the primary healthcare, the following recommendations are deduced from this study, also supported by the literature: i) theoretical learning should be accompanied by practical training, so that education is tied with real clinical practice [4], ii) an efficient model of outpatient consult liaison psychiatry, like the collaborative care model [31, 32] should be developed, that enhances the skills of GPs to manage psychiatric patients, and iii) adequate time for diagnosis, counselling, self-study and consultation should be given to GPs, along with incentives to be deeply involved in the management of mental disorders [4, 7, 15, 22, 33].

For an effective inclusion of mental health in the Primary Health Care, education should gain priority in healthcare systems applying long-term strategies. The level of education is reflected on correct diagnosis and successful treatment of CMDs and increases GPs' self-confidence. These are highly significant in a country, like Greece, which has suffered from an continuing financial crisis leading to high unemployment rates and social inequalities, in addition to fragmented health services which missed the chance to be fully reformed into a more community-oriented mental health system due to the recession, during the last years [32]. Recently, it has been reported that Greek psychiatry is developing in the right direction; however the public system of mental healthcare needs economic and organizational support, in order to complete the discontinued psychiatric reform [34].

### Limitations

This cross-sectional study focused on self-reported information given by the GPs, which bear the limitation of subjectivity and may differ from real data and records. Integral to this, the GPs' reported prevalence of CMDs reflects the rate of mental disorders' detection by them, rather than the true occurrence, assessed by specialists. In addition, there are factors that were not evaluated in this study, namely GPs' perceived role, limited time and level of discomfort in managing mentally ill persons [15, 32]. While the limited time that a GP can spend with a patient has been reported as an obstacle in almost all countries [20–22, 32, 33] and necessitates a healthcare system reform, the other two factors could be faced through CME [20].

## Conclusion

Management of mental disorders by GPs in Greece is amenable to improvement. Through the continuing medical education of GPs on mental health and development of liaison psychiatry in primary care, a successful integration of mental health into primary care could be achieved.

## Supplementary Information

**Additional file 1:** Pilot Survey. Ouestionnaire. **Table S1.** Demographic characteristics of general practitioners (GPs). **Figure S1.** Distribution of referrals of mental disorders cases by the GPs. **Table S2.** Mental diseases treated or referred by the GPs. **Figure S2.** GPs' preferred choices of continuing medical education on mental health. **Figure S3.** GPs' suggestions for facilitators on the management of patients with mental disorders, that should be provided by the healthcare system. **Figure S4.** Suggestions of GPs about successful integration of mental health into primary care.  

## Data Availability

The datasets used and/or analysed during the current study are available from the corresponding author on reasonable request.
