# Supplementary material for: General practitioners’ views towards management of common mental health disorders: Τhe critical role of continuing medical education
Source: BMC Prim Care. 2023 Mar 4;24:63. doi: 10.1186/s12875-023-02017-5 (PMC9985274; doi:10.1186/s12875-023-02017-5)
Supplement: Supplementary file 1 — Additional file 1: Pilot Survey. Ouestionnaire. Table S1. Demographic characteristics of general practitioners (GPs). Figure S1. Distribution of referrals of mental disorders cases by the GPs. Table S2. Mental diseases treated or referred by the GPs. Figure S2. GPs' preferred choices of continuing medical education on mental health. Figure S3. GPs' suggestions for facilitators on the management of patients with mental disorders, that should be provided by the healthcare system. Figure S4. Suggestions of GPs about successful integration of mental health into primary care. [file 12875_2023_2017_MOESM1_ESM.docx]

**Supplementary material**

**Pilot survey**

Thorough examination of the literature did not return a validated questionnaire addressing the aims of our study. A literature review and interviews with local experts in general practice and psychiatry helped the investigators select the survey items. Initially, a pilot survey was conducted in a small group of GPs and appropriate modifications of the questionnaire were made. Then, 40 GPs were contacted personally in the national conference of General Practitioners and asked to fill in a hard copy of the questionnaire. Subsequently, an electronic platform with the questionnaire was created and the same GPs were asked to fill in the questionnaire once again, after three months. The repeatability was estimated at 79% on average for the closed questions, while the Cronbach’s alpha coefficient of internal consistency was found to be 0.75, after appropriately reversing selected questions. The responses collected in the initial phase for the test-retest were not included in data processing.

**Questionnaire for General Practitioners on management of mental health disorders**

Gender Male Female Age: ______

Years of employment: ___

Employment in: Public sector: Health center ___ Regional Medical Centre ___ Hospital ___

Private sector: Private office ___ Private Clinic ___

*In the following sentences, please state what you personally believe about your current working situation, according to the following statements:*

*Choose only one of the options, the one that best describes your attitudes. Abbreviations are as follows: Completely Disagree (CD), Disagree (D), Rather Disagree (RD), Rather Agree (RA), Agree (A), Absolutely Agree (AA).*

*In questions 18 - 24 (open-ended) write your answer briefly in the space provided.*

1. I participate in psychiatric seminars/conferences with the aim of educating myself about mental disorders:

a. At least twice per year b. Once per year c. Every two years

d. Every three years e. Less often than once every 3 years f. Never

2. I participate in clinical training psychiatric courses to practice my skills on mental disorders:

a. At least twice per year b. Once per year c. Every two years

d. Every three years e. Less often than once every 3 years f. Never

3. I study scientific articles, contemporary research, and psychiatric textbooks, so that I can be informed about the most common mental disorders I am likely to meet in my practice.

a. Once a week b. Once a month c. Two - three times per year

d. Once per year e. Rarely f. Never

4. I get informed on the treatment of the most frequent mental disorders encountered in my practice, in terms of treatment regimens, pharmacodynamics and drug side-effects.

a. Once a week b. Once a month c. Two - three times per year

d. Once per year e. Rarely f. Never

5. My Continuing Medical Education (CME) in the field of psychiatry is adequate, so as to be able to respond to the needs of a person with a mental disorder CD D RD RA A AA

6. My diagnosis of a mental disorder is based on psychiatric disorder scales/psychometric tools (e.g., questionnaires, tests, etc.) CD D RD RA A AA

7. My diagnosis of a mental disorder is based on a psychiatric interview and the DSM-V criteria for mental disorders CD D RD RA A AA

8. How confident do you feel about diagnosis and treatment of a patient with a mental disorder? (1 indicates low confidence and 10 high confidence) 1 2 3 4 5 6 7 8 9 10

9. I am aware of the treatment that a patient should follow for a mental disorder I have diagnosed (pharmaceutical, psychological, psychoanalytical, combination) CD D RD RA A AA

10. After diagnosing a patient with a mental disorder, I refer him directly to a psychiatrist, without any medical intervention of my own, because he should be treated exclusively by a specialist doctor CD D RD RA A AA

11. After diagnosing a patient with a mental disorder, that requires medication, I initiate treatment, without referral to a psychiatrist CD D RD RA A AA

12. I follow-up a person with a mental disorder, only if there is guidance and precise instructions by a psychiatrist CD D RD RA A AA

13. I am aware of the mental health centers and services in my area, so as to be able to refer a patient, if needed CD D RD RA A AA

14. After referral to a psychiatrist, I make sure to be in contact and get informed about the diagnosis and possibly joint follow-up of the patient

a. Always b. Very often c. Often

d. Sometimes e. Rarely f. Never

15. I collaborate with psychiatrists/mental health centers, in order to consult them about my decisions CD D RD RA A AA

16. I am provided with all appropriate conditions to treat a patient with a psychiatric disorder, from diagnosis to recovery CD D RD RA A AA

17. After treatment initiation, if I do not experience the expected outcomes:

a. I modify my initial medication and wait for the outcome

b. I refer to a psychiatrist

c. Other

18. List in descending order the most common mental disorders you encounter in your practice

1.

2.

3.

4.

5.

6.

19. Which mental disorders do you undertake to deal with in your practice?

20. Which mental disorders do you refer to a psychiatrist/mental health service?

21. How many of the cases you see in your practice need to be referred to a psychiatrist/mental health specialist? (On a scale of 10) ...../10

22. What methods would you suggest for your continuing medical education (ECM) on mental health?

23. What would you like the healthcare system to provide in order to deal with patients with mental disorders? (this question applies only to those employed in the public sector)

24. What ways would you suggest in order to achieve an adequate and effective mental health integration into primary care?

**Table S1.** Demographic characteristics of general practitioners (GPs)

|  |  | **%**  **N = 355** | **Mean (range)** | **Standard Deviation** |
| --- | --- | --- | --- | --- |
| Gender | Male | 55.5 |  |  |
|  | Female | 44.5 |  |  |
| Age (years) |  |  | 45.0 (31.0-69.0) | 7.0 |
| Employed in | Private practice | 33.4 |  |  |
|  | Health Center | 28.9 |  |  |
|  | Regional Medical Center | 33.1 |  |  |
|  | Hospital | 4.5 |  |  |
| Work experience (years) | |  | 8.4 (0-35) | 6.7 |

Figure S1 depicts the distribution of referrals among GPs, on a percentage basis. It is observed that 39% of GPs stated they refer at least half of the cases they meet.

**

**

**Figure S1.** Distribution of referrals of mental disorders cases by the GPs

Table S2 presents the proportion of mental disorders that a GP refers to a specialist and those that a GP treats, in a descending order. These questions were open-ended, and GPs could write down up to six mental health disorders that they treat, and six that refer to a specialist.

**Table S2.** Mental diseases treated or referred by the GPs

| **Disorder** | **%** | **Disorder** | **%** | |
| --- | --- | --- | --- | --- |
| **Management by the GP** | | **Referral to the Specialist** | | |
| Mild / Moderate Depression | 84.4 | Psychotic Disorders | | 53.0 |
| Anxiety Disorders | 72.2 | No response to treatment | | 34.3 |
| Panic Disorders | 22.7 | Suicidal Ideation | | 25.5 |
| Organic Mental Disorders | 11.9 | Other | | 22.4 |
| Dementia | 9.6 | Bipolar Disorders | | 19.3 |
| Sleep Disorders | 7.9 | Severe / Complicated Disorders - Comorbidity | | 13.6 |
| Psychotic Disorders | 6.5 | Severe Depressive Episode | | 13.0 |
| Other | 6.5 | Uncertain Diagnosis | | 9.6 |
| Somatoform Disorders | 5.1 | Dependence Syndrome Depression | | 8.5 |
| Bipolar Disorders | 3.5 | Recurrent Mental Disorders | | 8.5 |
| None | 3.1 | All Disorders | | 7.1 |
| Phobic Anxiety Disorders | 2.7 | Mild / Moderate Depression | | 3.4 |
| Alcoholism-Dependence Syndrome | 1.4 |  | |  |
| Obsessive-Compulsive Disorder | 1.2 |  | |  |

Figure S2 presents GPs' proposals about ways of getting an improved education on mental health. Figure S3 depicts GPs' responses on what facilitators would they like to be provided by the health care system, in order to be able to deal with a mentally ill person successfully, and Figure S4 illustrates their suggestions regarding successful integration of mental health into primary care.

**Figure S2.** GPs' preferred choices of continuing medical education on mental health. Blue bars present theoretical learning methods, while orange ones depict practical training methods.

**Figure S3.** GPs' suggestions for facilitators on the management of patients with mental disorders, that should be provided by the healthcare system

**Figure S4.** Suggestions of GPs about successful integration of mental health into primary care
